# The Completeness of the Fossil Record of Mesozoic Birds: Implications for Early Avian Evolution

**DOI:** 10.1371/journal.pone.0039056

**Published:** 2012-06-25

**Authors:** Neil Brocklehurst, Paul Upchurch, Philip D. Mannion, Jingmai O'Connor

**Affiliations:** 1 Museum für Naturkunde, Leibniz-Institut für Evolutions und Biodiversitätsforschung, Berlin, Germany; 2 Department of Earth Sciences, UCL, London, United Kingdom; 3 Department of Earth Science and Engineering, Imperial College London, London, United Kingdom; 4 Institute of Vertebrate Paleontology and Paleoanthropology, Beijing, China; 5 Dinosaur Institute, Natural History Museum of LA County, Los Angeles, California, United States of America; College of the Holy Cross, United States of America

## Abstract

Many palaeobiological analyses have concluded that modern birds (Neornithes) radiated no earlier than the Maastrichtian, whereas molecular clock studies have argued for a much earlier origination. Here, we assess the quality of the fossil record of Mesozoic avian species, using a recently proposed character completeness metric which calculates the percentage of phylogenetic characters that can be scored for each taxon. Estimates of fossil record quality are plotted against geological time and compared to estimates of species level diversity, sea level, and depositional environment. Geographical controls on the avian fossil record are investigated by comparing the completeness scores of species in different continental regions and latitudinal bins. Avian fossil record quality varies greatly with peaks during the Tithonian-early Berriasian, Aptian, and Coniacian–Santonian, and troughs during the Albian-Turonian and the Maastrichtian. The completeness metric correlates more strongly with a ‘sampling corrected’ residual diversity curve of avian species than with the raw taxic diversity curve, suggesting that the abundance and diversity of birds might influence the probability of high quality specimens being preserved. There is no correlation between avian completeness and sea level, the number of fluviolacustrine localities or a recently constructed character completeness metric of sauropodomorph dinosaurs. Comparisons between the completeness of Mesozoic birds and sauropodomorphs suggest that small delicate vertebrate skeletons are more easily destroyed by taphonomic processes, but more easily preserved whole. Lagerstätten deposits might therefore have a stronger impact on reconstructions of diversity of smaller organisms relative to more robust forms. The relatively poor quality of the avian fossil record in the Late Cretaceous combined with very patchy regional sampling means that it is possible neornithine lineages were present throughout this interval but have not yet been sampled or are difficult to identify because of the fragmentary nature of the specimens.

## Introduction

Birds (Aves) represent one of the most diverse and abundant vertebrate groups, with over 10,000 species [1] and an estimated 300 billion individuals alive today [2]. The avian fossil record extends back to the Late Jurassic, or possibly further [2] (although see [3] and [4] and Materials and Methods below). This fossil record, in particular that of the Mesozoic, has recently undergone a revolution as a result of an explosion of newly discovered taxa during the last three decades. At present, over 120 avian species are known from the Mesozoic, from all continents except mainland Africa. Despite this new information, controversy surrounds several aspects of avian evolution, including the timing of the origin and diversification of modern birds (Neornithes).

**Figure 1:**
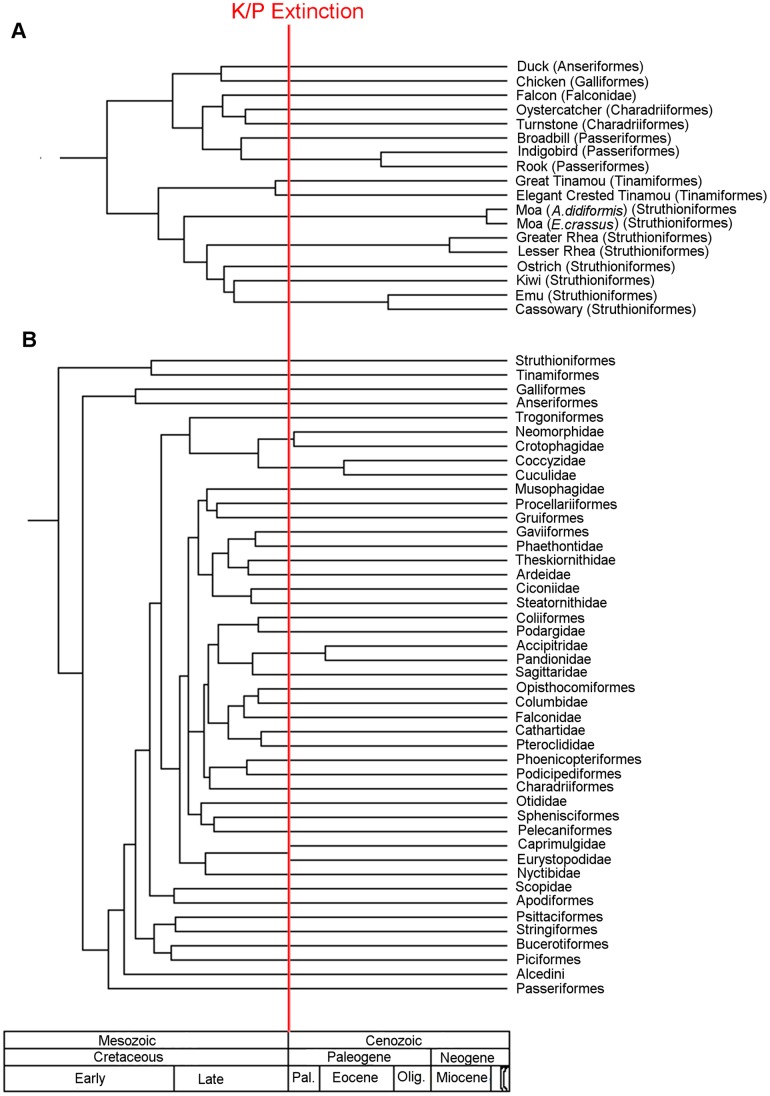
The two opinions on the timing of modern bird origins based on molecular clocks. A) an origin 123 million years ago during the Aptian, modified from reference [11]; B) an origin 135 million years ago during the Valanginian modified from reference [13].

Much of the debate surrounding neornithine evolution focuses on the apparent discrepancy between the time of their origins according to molecular data and their earliest appearance in the fossil record. The ‘traditional’ view of neornithine origins envisaged the evolution of modern groups in the Cretaceous [5]–[7]. This was based on the assignment of numerous species of Mesozoic bird fossils to extant orders [8], such as the placement of the Hesperornithiformes (toothed aquatic birds with reduced forelimbs from the Cretaceous) in a clade containing loons and grebes [6], even though this requires an evolutionary reversal to the plesiomorphic toothed condition [9]. Several studies based on molecular clocks support the traditional view of gradual neornithine diversification starting in the Early Cretaceous [10]–[13]. The exact timing of these events varies with each analysis; Kumar & Hedges [10] and Paton et al. [11] suggested that Neornithes originated during the Aptian (125–112 million years ago [mya]), whereas Cooper & Penny [12] and Brown et al. [13] proposed an origin as early as the Valanginian (140–133 mya) (Figure 1). Biogeographic analysis has also supported the idea of a Cretaceous origin: for example, Cracraft [14] found neornithine evolution to have been heavily influenced by vicariance (the isolation of lineages by the splitting of continents), and suggested that they diversified with the breakup of Gondwana during the Cretaceous [15]. The divergence of the majority of neornithine clades during the Cretaceous would suggest that the Cretaceous/Palaeogene (K/Pg) mass extinction had relatively little effect on this group, although Feduccia [16] considered this unlikely, since birds are often extremely sensitive to environmental perturbations.

In contrast, many recent palaeobiological, and some molecular, studies have disputed such an early origin of Neornithes. Hope [17] documented 50 putative Cretaceous neornithine specimens from as early as the Coniacian, but these are all extremely fragmentary and none were subjected to formal phylogenetic analysis. The characters used to assign them to neornithine clades are often dubious or incorrect (see [18] for a summary), and attempts to place these fossils in modern groups have been hindered by poor knowledge of neornithine relationships [19]. There has been relatively little morphological analysis of modern clades incorporating their fossil representatives, and as such the relationships between the taxa and the characters that unite them are not well understood. Benton [20] argued that all records of Neornithes prior to the K/Pg boundary are either misdiagnosed or are from incorrectly dated localities; for example the Hornerstown Formation in the USA, originally dated as latest Maastrichtian and containing several putative neornithine species, has been reinterpreted by some as being from the earliest Palaeocene [21], although this remains uncertain [17]. A phylogenetically based study of the avifauna of the Hell Creek, Lance and Frenchman Formations of North America (the only formations containing bird specimens that can be reliably dated to the end of the Maastrichtian) [22] found no compelling evidence for a neornithine radiation prior to the K/Pg boundary. Instead, the majority of the birds were found to be more basal ornithurines, with three enantiornithines, none of which extend into the Palaeogene [22] (see also [23]).

Since the summary presented by Hope, more Mesozoic specimens have been assigned to the Neornithes. A coracoid, found in beds of Turonian–Coniacian age in Patagonia, was described as a galliform [24], while a quadrate originally assigned to *Cimolopteryx rara* from the Lance Formation [25], of late Maastrichtian age, was re-described as an anseriform [26]. A left carpometacarpus from the Campanian–Maastrichtian Allen Formation of Argentina has also been described as cf. Neornithes [27]. However all three of these specimens each consist of only a single bone, and their tentative assignments to neornithine clades were based on general comparisons rather than cladistic analysis. *Teviornis gobiensis*, another putative anseriform from the Maastrichtian Nemegt Formation of Mongolia [28], is better known, being represented by a complete forelimb, but again no formal cladistic analysis has been carried out, and its assignment to the Neornithes has been questioned [29]. In contrast, *Vegavis iaai* from the latest Cretaceous of Antarctica [30] has been subjected to phylogenetic analysis which supported a position within the Anseriformes. While this discovery pushes the neornithine record as far back as the Maastrichtian, this is still considerably later than is suggested by most molecular clock studies (see above).

**Figure 2:**
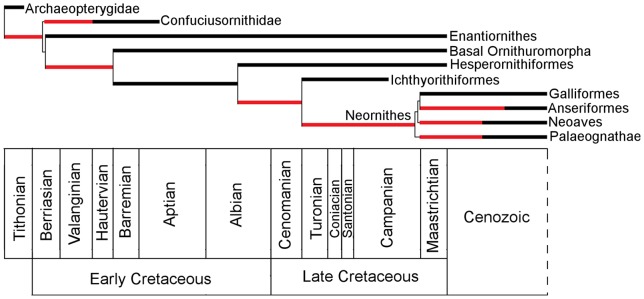
Neornithine evolution based on a ‘literal’ interpretation of the fossil record. According to this scenario of avian evolution, the Neornithes did not appear until the latest Cretaceous, and then diversified rapidly in the Cenozoic, whereas all basal bird groups died out at or before the K/Pg boundary. Black lines represent lineages present in the fossil record, red lines represent ghost lineages inferred from phylogenetic analysis.

Thus, the current palaeobiological perspective on these events is that most basal bird lineages suffered a catastrophic extinction at the K/Pg boundary, whereas the Neornithes originated in the latest Cretaceous and radiated in the early Cenozoic [14], [20], [30], [31] (Figure 2). This is supported by the molecular clock study of Ericson et al. [32], based on nuclear genes, which found that most extant avian lineages diverged after the K/Pg boundary, with only a few basal lineages appearing a short time before the end-Cretaceous mass extinction (although Ericson et al.'s results have been criticised because of poor choices of fossil calibrations and a lack of error bars around divergence time estimates [33]).

One way to reconcile the current fossil record of Mesozoic birds with the molecular clock-based Cretaceous divergence times for Neornithes is to propose that members of this clade were present earlier in the Cretaceous but that their fossil record is currently too incomplete for them to be unequivocally recognised by palaeobiologists. For example, Kumar & Hedges [10] implied that the reason the fossil record has not produced reliable Cretaceous neornithines is that birds are small animals with delicate skeletons, and therefore not easily preserved. However, palaeobiologists have not found this to be a compelling argument. In particular, Benton [20] noted that other animals with delicate skeletons, including basal birds, lizards, amphibians and small mammals, have been found throughout Cretaceous sediments, so their preservation is certainly possible. However, it is conceivable that a sampling bias or other taphonomic factor might have selectively preserved basal birds relative to Neornithes. For example, such a selective mechanism might have operated if basal bird species were much more abundant than early Neornithes: this is because population size might correlate with the probability of individuals making it into the fossil record. Another such bias might occur if neornithines originated and diversified in a part of the world that has a particularly poor avian fossil record, such as Africa. Finally, it is possible that neornithines showed a preference for different environments to basal birds and other small bodied taxa, and that these habitats varied in terms of their potential to preserve fossils [34].

Issues relating to the sampling and quality of the Cretaceous avian fossil record are clearly central to resolving the current discrepancies between molecular clock and palaeobiological estimates for the timing of the neornithine radiation. Several previous studies have examined the quality of the fossil record of Cretaceous birds, in order to estimate how much of the record might be missing. Fara & Benton [35] applied the simple completeness metric, which is a measure of the proportion of Lazarus taxa relative to observed taxa in the fossil record. These authors found a simple completeness metric value of 77.4% for Mesozoic birds, a high level of completeness relative to other vertebrate groups such as amphibians and squamates [35], implying a large proportion of the early avian fossil record is known. Bleiweiss [36] used gap analysis to estimate the extent to which three neornithine lineages can be extrapolated back in time beyond their first fossil occurrences, based on the number and length of ‘gaps’ in their fossil record. No support for a missing fossil record stretching far back into the Cretaceous was found. However, analysis of three clades represents only a small proportion of extant neornithine diversity. The accuracy of this technique also depends on the earliest known representative of a particular clade being identified and dated correctly, and this is clearly problematic for the earliest Neornithes (see above).

Fountaine et al. [37] used a different approach to assess the quality of the fossil record of Mesozoic birds. They quantified the completeness of each individual avian species, grading each on a scale of 1–4, whereby a species given a grade of 1 was represented by a single bone, 2 by more than one bone, 3 by a single nearly complete specimen and 4 by more than one nearly complete specimen. These completeness scores were then used to summarise the overall quality of the avian fossil record for time bins throughout the Mesozoic. It was found that most Cretaceous time bins had a ‘good’ fossil record (i.e. species graded 3 and 4 either out-numbered or were of similar frequency to those graded 1 and 2), except for the Maastrichtian, when fragmentary species were more common [37]. It was also argued that basal bird phylogeny was largely congruent with the sequence of appearance of clades in the fossil record, suggesting that there was little missing data [37]. Finally, the idea that the quality of the avian fossil record decreases with increasing stratigraphic age was disputed based on the observation that the number of bird-bearing localities and the number of described species are random with respect to geological age [37]. As such Fountaine et al. [37] deemed the avian fossil record of high enough quality to determine genuine biological signals. Consequently the poor completeness of putative Cretaceous neornithines was judged to represent their rarity at this time rather than any geological bias.

There are some drawbacks with the method used by Fountaine et al. to quantify specimen and species completeness (see ‘Methods and Materials’ below), and a re-evaluation of the Mesozoic avian fossil record is timely given the recent influx of new data (28 new species since Fountaine et al.'s study). In the current work, therefore, we present an updated and highly revised data set of Mesozoic birds (124 valid species), and assess fossil record quality using a recently developed character completeness metric [38]. We assess the impact of fossil record quality on the taxic diversity of Mesozoic birds by comparing completeness scores with observed diversity and a sampling-corrected diversity estimate. The impact of new discoveries is explored by comparing our updated dataset with that of Fountaine et al. [37]. Factors that might control or bias the quality of the avian fossil record are assessed by comparing completeness scores with a sea level curve, and by evaluating how fossil record quality varies with depositional environment and latitude. We also test whether the fossil record of small delicate organisms (birds) is better or worse than that of large robust forms (sauropodomorph dinosaurs). To conclude, we examine the implications of these analyses for claims concerning the presence/absence of neornithine fossils prior to the K/Pg boundary.

## Materials and Methods

### Dataset

Data on the occurrences of all Mesozoic bird species were compiled from the published literature as well as the Paleobiology Database (PBDB: www.paleodb.org) and were then scrutinized for synonyms and *nomina dubia*. *Archaeopteryx* is here considered to be a bird, despite one recent phylogenetic study [39] that placed it closer to dromaeosaurs than to Aves (a further analysis, using the same dataset but applying a maximum likelihood analysis method, returned *Archaeopteryx* to Aves [40]). Since no published description of *Proornis coreae* exists, this species is a *nomen nudum* and excluded from the analysis. *Rahonavis ostromi* [41] was excluded following phylogenetic analyses [42]–[45] which recovered it as a non-avian theropod. The Late Triassic taxon *Protoavis texensis*, of dubious avian affinity [3], [4], was also not included, so the time period under study stretches from the Tithonian to the end of the Maastrichtian (150.8 to 65.5 Mya). The final dataset (see Table S1) consists of the stratigraphic ranges, geographic distributions and character completeness metric scores (see below) for the 124 valid avian species in 82 genera. This dataset can be regarded as up-to-date as of May, 2011.

We have also used data on six other parameters: (1) the number of specimens named to species level in the literature per geological substage; (2) the environment in which the birds were preserved (data from the published literature and the PBDB); (3) the number of fluviolacustrine bird-bearing localities (data from the published literature and the PBDB); (4) the number of theropod-bearing collections per geological substage (data from the PBDB) (5) the number of dinosaur bearing formations per geological substage (6) the completeness metric values of Sauropodomorpha (data from reference [38]) and (7) sea level (data from reference [46]).

### Completeness Metrics

The specimen completeness scoring systems proposed by Fountaine et al. [37] for Mesozoic birds and the similar method used by Benton [47] for dinosaurs are problematic because they are somewhat subjective and provide only coarse-grained quantifications of specimen quality. For example, where exactly is the boundary between a collection of associated skeletal elements (scored as ‘2’ in Fountaine et al.'s scheme) and a nearly complete skeleton (scored as ‘3’)? Different workers could assign different completeness scores to the same specimens, making it difficult to reproduce the results of analyses of specimen completeness. Also, the coarse-grained nature of completeness metrics based on just four or five categories means that important fluctuations in fossil record quality might be obscured. For these and other reasons, Mannion and Upchurch [38] introduced a fine-grained and more objective basis for assessing specimen and taxon completeness. These completeness metrics include: (1) the ‘Skeletal Completeness Metric’ (SCM) which attempts to capture the completeness of a specimen or taxon on the basis of the relative bulk and number of the elements that are preserved; and (2) the ‘Character Completeness Metric' (CCM) which quantifies the amount of phylogenetically relevant information preserved in a specimen or taxon. In this study, we only apply the CCM because this is regarded as being more appropriate than the SCM for studies of the relationship between fossil record quality and taxic diversity [38].

In order to estimate the CCM for each species, it is necessary to obtain a list of phylogenetic characters for the group under study. Here, a list of such characters was compiled based on three phylogenetic analyses of basal birds [48]–[50], one of neornithine birds [51] (only osteological characters used), and one of coelurosaurian theropods [45]. These separate character sets were combined and duplicate characters were removed, leaving a list of 655 characters (see Table S2). The number of characters pertaining to different parts of the skeleton differs for coelurosaurs+birds relative to those of sauropodomorphs (see Table 1), but in both groups the complex cranial anatomy means that this region of the skeleton is responsible for a disproportionately large number of phylogenetic characters.

**Table 1:** The percentage of characters relating to each skeletal region in Aves and Sauropodomorpha (data on Sauropodomorpha from reference [33]).

Skeletal Region	Aves	Sauropodomorpha
Skull	32.37%	33.49%
Vertebral Column and Ribs	11.76%	25.78%
Pectoral Girdle	11.38%	3.19%
Forelimbs	19.39%	11.63%
Pelvic Girdle	10.76%	7.89%
Hindlimbs	16.18%	18.15%
Integument	0.46%	N/A

Mannion & Upchurch [38] proposed two ways of implementing the CCM. The first, CCM1, estimates the completeness of the most complete specimen of each species. The second, CCM2, assesses completeness based on the combined information from all specimens assigned to a species [38]. CCM2 was considered to be a more meaningful measure than CCM1, because it estimates the total information available from all known specimens rather than simply from the best preserved individual [38]. For example, if one specimen preserves the skull and neck region, and another preserves the neck and forelimbs, then CCM2 estimates the completeness of the taxon based on the characters that can be scored for the skull, neck and forelimbs. Here, CCM2 is preferred over CCM1 because the latter requires some species to be omitted from an analysis in cases where associations of disarticulated bones make it difficult to recognise ‘the most complete individual’ (e.g., as occurs in many bone bed deposits [38]). In any case, the choice of completeness metric might not be critical: Mannion & Upchurch [38] found strong positive correlations between all of the various metrics (SCM1, SCM2, CCM1, CCM2) for sauropodomorph dinosaurs.

The CCM2 score for a given Mesozoic avian species has been calculated as follows. Each element or portion of element preserved in a specimen can be scored for a given number of the total characters available. For example, a complete, well-preserved maxilla can be scored for seven characters (i.e., 1.07% of the 655 skeletal characters that can be scored for coelurosaurs+birds). Thus, if an extinct avian species is known solely from a complete maxilla, then the CCM2 for that species is 1.07%. If, however, a second specimen of this species is known, and if that specimen preserves a maxilla and a femur (the latter being scorable for 2.6% of the characters), then the CCM2 for the species is 1.07%+2.6% = 3.67%. In other words, the CCM2 score for a species is the percentage of characters that can be scored for that taxon in the character list.

The characters referring to the elongated bones of the pectoral and pelvic girdles and the fore and hind limbs were divided into sets that pertain to the ‘proximal end’, ‘distal end’, and ‘shaft’. Unlike Mannion & Upchurch's analysis of sauropodomorphs [38], the contribution of each skull element and each manual and pedal digit was scored individually. For each section of the vertebral column (cervical, thoracic, sacral and caudal), the characters were divided into four sets depending on whether they can only be scored when a single vertebra, an anterior vertebra, a posterior vertebra, or the entire series, is preserved. Because the neural spines are missing from the vertebrae in many specimens, characters pertaining to the neural spines were coded separately in each section of the vertebral column. Table S3 presents a complete list of the percentage contributions to the CCM2 made by each skeletal element or part of an element.

### Data bins and average CCM2 scores

The quality of the avian fossil record is represented by taking the mean CCM2 score for all species occurring within a given time bin. These averages can then be plotted through time, providing a depiction of how avian fossil record quality fluctuated during the Mesozoic (see below). The standard deviation around the mean was also calculated. The time bins used were the geological stages of the Mesozoic. Each stage (the timescale provided by reference [52]) was divided into ‘early’ and ‘late’ substages with the boundary at the midpoint of the stage. When different specimens of one species occur in different substages, they were treated separately. If a specimen was of uncertain age, and could not be resolved to a particular substage, it was included as present in the entire range of substages to which it might have belonged. Table S1 presents a list of all Mesozoic avian species included in the analysis, the time period to which they were assigned and their completeness scores. The same time bins have been used in the plots of raw taxic and residual diversity for Mesozoic birds, the CCM2 scores for sauropodomorphs, and the sea level curve (an interpolated version of the curve from reference [46] presented by Butler et al [53], calculating a mean average for each substage). Additionally, Mesozoic avian species were sorted by the modern latitude of the locality at which they were found. The mean completeness score for all the birds in each 5° latitudinal bin was calculated.

One problem with assessments of fossil record quality based on mean CCM2 scores is that these values might be strongly affected by sample size. A time or latitudinal bin that has yielded only a small number of specimens might have a very low mean CCM2 or very high mean CCM2 by chance, merely because the first few specimens to be found happen to be relatively incomplete or complete respectively. Such variation in mean CCM2 would not provide a very meaningful way of assessing differences in the general level of specimen completeness between data bins. Ideally, this issue should be addressed via, for example, a subsampling approach that would randomly select a specified number of specimens or species from each data bin. Unfortunately, this is not practical with regard to the current Mesozoic avian fossil record because of the relatively low numbers of species and specimens in many data bins. Such low sampling means that, either many data bins would have to be omitted from the subsampling analysis, or extremely low subsample sizes would have to be imposed on all data bins (the latter tending to result in artefactual dampening of fluctuations in completeness scores across data bins). Here, therefore, we show numbers of avian specimens appearing in the literature representing valid species in each temporal and latitudinal bin, alongside mean CCM2 scores, so that the reader can see which data bins are relatively well or poorly sampled. Conclusions based on data bins with particularly low sample sizes should be treated with caution, and such issues are highlighted in the relevant sections of the discussion.

### Residual diversity estimate (RDE)

The taxic diversity counts for Mesozoic birds have been ‘corrected’ for potential sampling biases using the residuals method of Smith and McGowan [54]. This approach first organises the taxic diversity counts and a sampling metric into two data series so that each has its values ranked from low to high. The number of theropod (both avian and non-avian)-bearing collections (any collection [e.g. a quarry] containing theropod material) is used as the sampling metric in this case. This proxy is used since it shows a significant positive correlation with the taxic diversity, and also to address two criticisms raised against the use of proxies such as fossil-bearing collections and formations. The first is that they are redundant with diversity: if the diversity of birds decreased, one would expect a lower number of bird-bearing formations or collections [55], [56]. This problem may be mitigated by using a sampling proxy based on a group more inclusive than, but containing, the group under study [57], [58]. If the diversity of birds decreased, the diversity of other theropods would not necessarily show the same decrease, so one would not expect the number of theropod-bearing collections to decrease. The second criticism is that such proxies do not take into account non-occurrences i.e. instances where workers have looked for fossils in formations, but not found them [56]. Again, using a proxy based on a larger group that includes the group under study mitigates this concern. The number of theropod-bearing collections includes instances when searches have been made in rocks containing species closely related to birds, but no birds have been found.

All data were log transformed prior to the calculation of regression equations and statistical testing (to allow values of 0 to be log transformed, 1 was added to every value). A regression equation which expresses the relationship between these data series is then calculated: this equation represents a ‘model’ of the relationship between sampling and observed diversity in the fossil record. Residual diversity values are then calculated by subtracting the predicted diversity from the observed diversity (i.e., residuals represent the amount of diversity that cannot be explained by sampling) [53], [54], [58]–[63]. Confidence intervals were placed around the residual diversity using the standard deviations of the model, following the method of Lloyd [64].

### Statistical tests

Two statistical tests were used to compare the time series of mean CCM2 scores to various other parameters. The Spearman's rank correlation coefficient is a non-parametric measure of how two variables are ordered [65]. Kendall's tau rank correlation coefficient is also a non-parametric statistic, which measures whether two curves change synchronously [65]. Generalised differencing was implemented to correct for trend and autocorrelation [65], [66]. To compare the completeness of bird specimens found in different environments, and the completeness of birds to that of sauropodomorphs, the Mann Whitney U-test was used. This is a non-parametric test comparing the medians and standard deviations of two data sets [65]. Statistics were executed using the computer program PAST [67].

## Results

### Avian fossil record completeness

**Figure 3:**
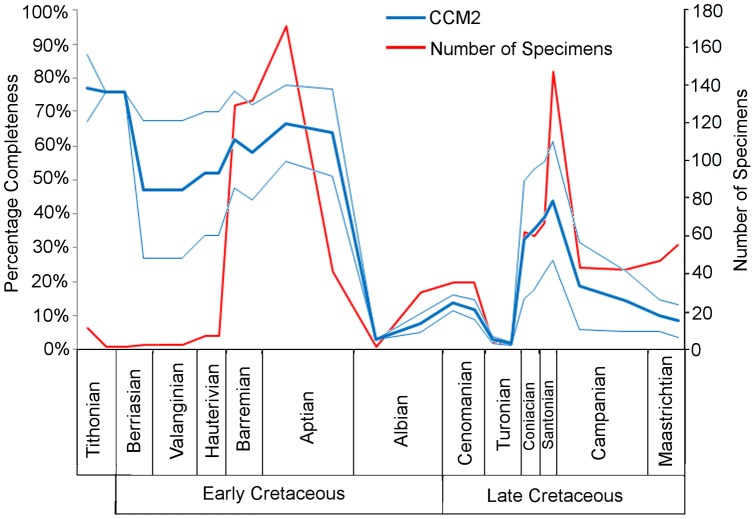
The mean character completeness metric scores for all Mesozoic birds in each substage. The bold blue curve represents the mean CCM2 scores, while the thin blue lines above and below it represent one standard deviation above and below the mean. Numbers of specimens are shown by the red curve to indicate sample size.

The mean CCM2 scores for each substage are plotted against time in Figure 3, along with their standard deviation and the number of specimens. Completeness levels are at their highest (76.84%) during the earliest substage (early Tithonian). Completeness remains high during the late Tithonian and early Berriasian, with only one taxon present in these bins (*Shenqiornis mengi* [68]; see ‘Discussion’ for further comments on how the uncertain date of this specimen may have affected the results). Following this, there is a decrease in completeness to 46.95% in the late Berriasian. Values start to rise again during the early Hauterivian, and continue to rise to a second peak of 66.50% in the early Aptian. The values decline slightly in the late Aptian, and at the Aptian/Albian boundary fall to 9.16%. The values remain between 0 and 20% for the rest of the Late Cretaceous, apart from a brief peak in the Coniacian and Santonian (the highest completeness score in the Late Cretaceous is 43.72% in the late Santonian, whereas the lowest is 1.53% in the late Turonian).

### Comparisons with Fountaine et al. [37]

**Figure 4:**
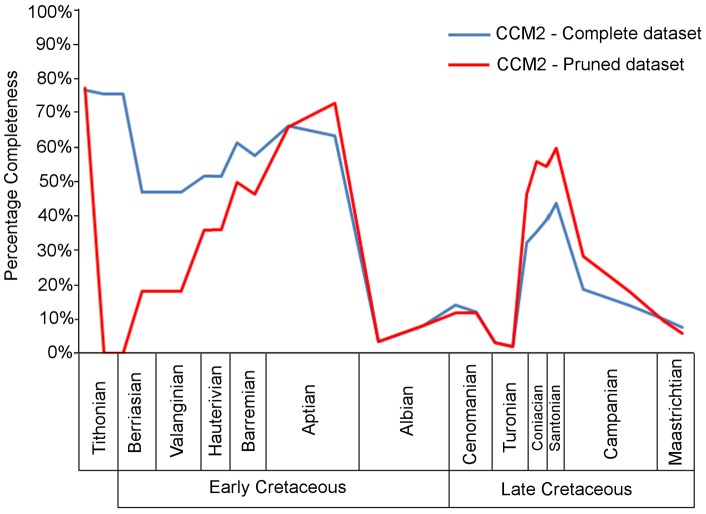
The CCM2 scores for Mesozoic birds, using both the complete dataset and the pruned dataset. The complete dataset (blue curve) includes all 124 species, the pruned dataset (red curve) includes only species known to Fountaine et al [37].

The study of the quality of the avian fossil record by Fountain et al. [37] employed a different method for quantifying completeness (see above) and (inevitably given its earlier date of publication) a smaller dataset than that used herein. These differences provide an opportunity to investigate: (1) the impact of the influx of new data on Mesozoic birds during the past eight years (N.B. the dataset of Fountaine et al. was finalised in 2003); and (2) the extent to which conclusions regarding fossil record quality might vary depending on the method used to estimate completeness. The first issue is addressed by measuring the correlation between CCM2 values based on all currently known Mesozoic bird species and the CCM2 values for only the species available to Fountaine et al. (i.e. a pruned version of our dataset). The result (Table 2) demonstrates the presence of a strong positive correlation between these two CCM2 time series. The only periods in which the two curves (figure 4) differ greatly are the late Tithonian and early Berriasian. Since the only bird included in the dataset from these substages is the well preserved *Shenqiornis mengi* (although again the uncertain date of this taxon should be noted; see ‘Discussion’), the completeness score for this substage in the complete dataset is 75.72%. However, this species was described after the study of Fountaine et al, so the completeness score for the late Tithonian and early Berriasian in the pruned dataset is 0%. Between the late Berriasian and the Aptian, the two curves show the same upward trend, although the complete dataset maintains a higher average completeness than the pruned dataset. Elsewhere the curves show the same peaks and troughs, although in some cases the height of these differs: the pruned dataset shows a higher completeness of species from the Coniacian, Santonian and Campanian than the complete dataset.

**Table 2:** Kendall's tau and Spearman's rho correlation coefficients and uncorrected probability values (*p*) of the statistical comparisons of time series after generalised differencing.

Statistical Test	Kendall's tau	Spearman's rho
Theropod-bearing collections vs taxic diversity	0.38 (*p* = 0.007757)	0.52538 (*p* = 0.0069971)
Dinosaur-bearing formations vs taxic diversity	0.32923 (*p* = 0.018352)	0.44889 (*p* = 0.021432)
Mean CCM2 (Current dataset) vs mean CCM2 (taxa used by Fountaine et al. [32])	0.45231 (*p* = 0.0011948)	0.58154 (*p* = 0.0018333)
Mean CCM2 vs mean grades used by Fountaine et al. [32]	0.32308 (*p* = 0.020648)	0.47966 (*p* = 0.013152)
Mean CCM2 vs taxic diversity	0.36 (*p* = 0.0099127)	0.45505 (*p* = 0.019504)
Mean CCM2 vs residual diversity	0.39077 (*p* = 0.0051217)	0.55145 (*p* = 0.0039007)
Mean CCM2 (Aves) vs mean CCM2 (Sauropodomorpha)	−0.21231 (*p* = 0.12829)	−0.333265 (*p* = 0.096826)
Mean CCM2 (Aves) vs number of bird-containing fluviolacustrine localities	0.24308 (*p* = 0.081634)	0.33128 (*p* = 0.09829)
Mean CCM2 (Aves) vs sea level	−0.021538 (*p* = 0.87738)	0.0092308 (*p* = 0.9643)

**Figure 5:**
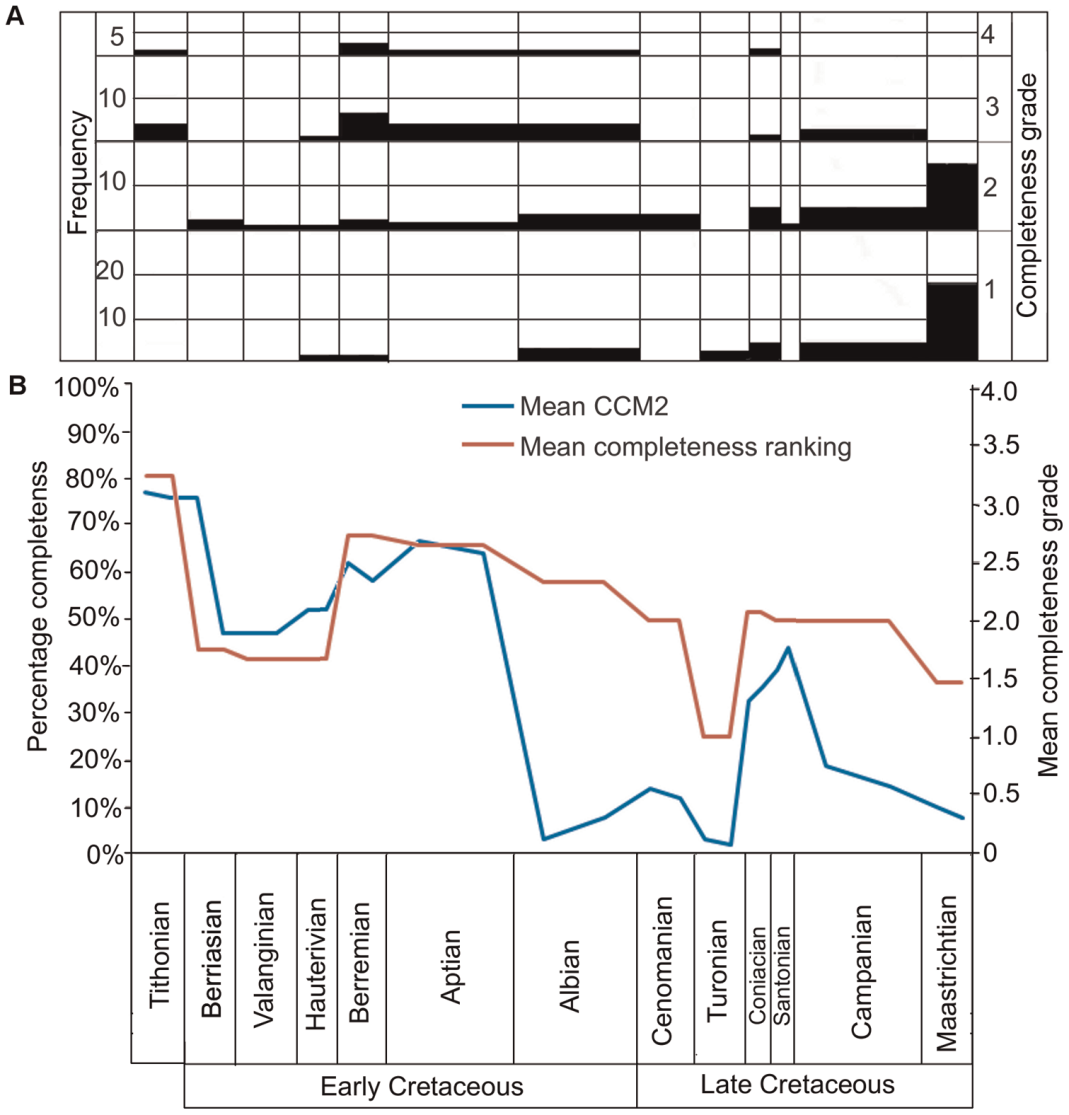
Comparison of the data collected by Fountaine et al. [37] with the CCM2. (A) Modified from Fountaine et al.'s [37] assessment of the completeness of the fossil record of Mesozoic birds (bird species with a completeness grade of 1 are represented by one bone, those with 2 by more than one bone, those with 3 by a nearly complete specimen, and those with 4 by more than one specimen); (B) Comparison of the mean completeness grade of bird species using the method of Fountaine et al. (red curve) and the mean CCM2 scores determined in this study (blue curve).

The impact of the choice to use the 1–4 completeness grading system of Fountaine et al. [37], versus the CCM2, has been examined by calculating the mean completeness grade for all species in each substage (Figure 5). Comparison of these averaged completeness grades with CCM2 recovered a significant positive correlation (Table 2). However this correlation is not as strong as that between the CCM2 including the complete dataset and the pruned CCM2 including only species available to Fountaine et al. (Table 2). Thus it is clear that any differences in interpretation between the results presented here and those presented in Fountaine et al. [37] probably reflect the effects of choice of methodology rather than the addition of new data. The process of grading the specimens from 1 to 4 does produce noticeable differences in estimated fossil record quality compared to that based on mean CCM2 (see ‘Discussion’).

### Avian diversity and fossil record completeness

**Figure 6:**
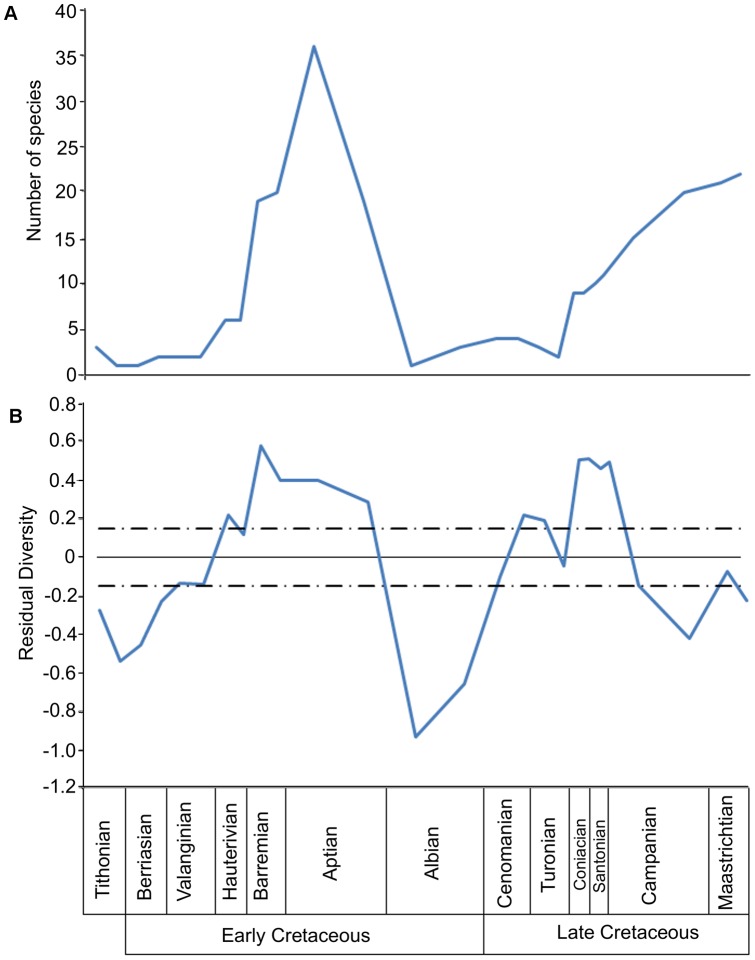
Two estimates of Mesozoic avian diversity. (A) the raw taxic diversity estimate; (B) The residual diversity curve corrected for the number of theropod-bearing collections (dashed-dotted lines indicate standard deviation from the model).

Raw (uncorrected) taxic diversity and our ‘sampling-corrected’ residual diversity estimates for Mesozoic birds are shown in Figure 6. The sampling corrected residual diversity curve was based on the number of theropod-bearing collections (see ‘Materials and Methods’). There is a statistically significant positive correlation between the taxic diversity of birds and our completeness scores and an even stronger correlation between the latter and the residual diversity estimate (Table 2).

### Controls on avian fossil record completeness

#### Sea level

**Figure 7:**
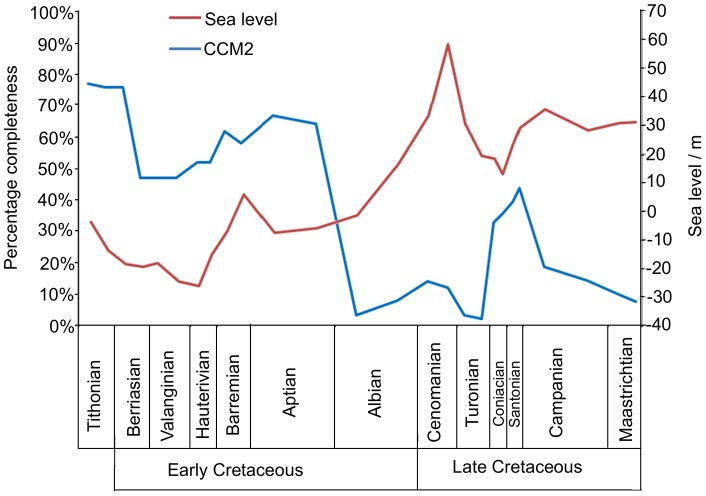
A comparison of sea level [53] and the mean CCM2 scores of Mesozoic bird specimens.

The correlation between the mean CCM2 values and the sea level curve of Miller et al. [46] (Figure 7) is weak and statistically non-significant (see Table 2).

#### Depositional environment

**Figure 8:**
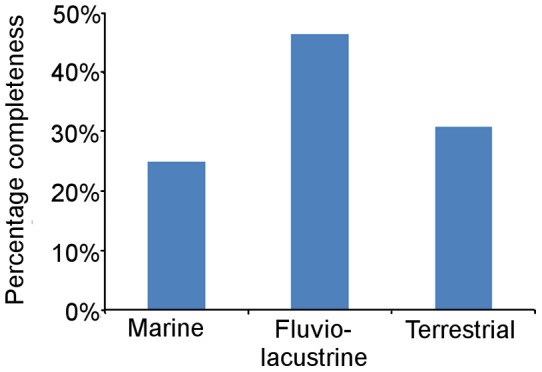
The mean CCM2 scores for birds found in three different palaeoenvironments: marine, fluviolacustrine, and non-fluviolactustrine terrestrial evnvironments.

**Figure 9:**
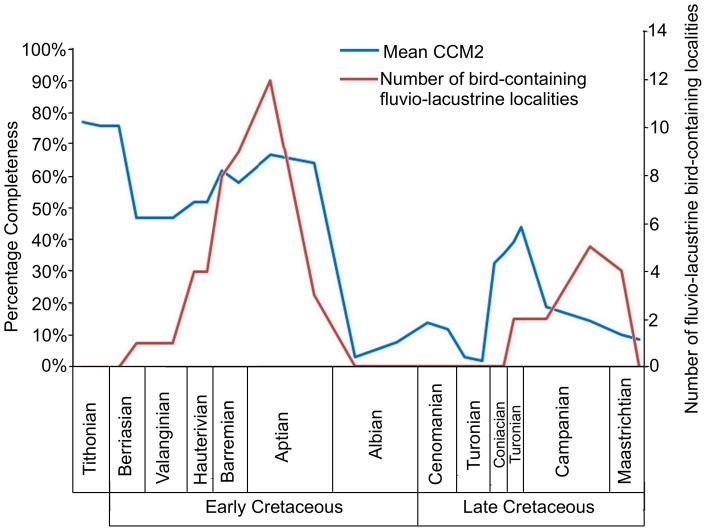
The number of fluvialolacustrine bird-bearing localities in each substage compared to mean CCM2.

The localities that have yielded Mesozoic bird specimens were divided into three environmental categories: marine, fluviolacustrine, and non-fluviolacustrine terrestrial environments. The CCM2 scores for taxa from each type of environment were then compared using the Mann-Whitney U-test. The mean completeness scores of birds from fluviolacustrine localities were much higher than those found in the other environments (Figure 8). The Mann-Whitney test suggests that the completeness of birds from fluviolacustrine environments is significantly greater than that of species from the other two categories (Table 3). The CCM2 of birds from marine and non-fluviolacustrine terrestrial localities are more similar to each other, with no significant difference according to the Mann-Whitney test (Table 3). The mean CCM2 scores were then compared to the number of fluviolacustrine localities in each substage time bin (Figure 9) but there is no significant correlation between these two parameters (Table 2).

**Table 3:** Mann-Whitney U-Test Scores and for the probability values of equality of medians (*p*), for comparisons of CCM2 scores of birds and sauropods, and the CCM2 scores of birds from different environments.

Statistical Test	Mann-Whitney U Score
Mean CCM2 of birds from fluvialolacustrine localities vs mean CCM2 of birds from other terrestrial localities	U = 587 (*p* = 0.01273)
Mean CCM2 of birds from fluvialolacustrine localities vs mean CCM2 of birds from marine localities	U = 505 (*p* = 0.004826)
Mean CCM2 of birds from marine vs mean CCM2 of birds from non-fluviolacustrine terrestrial localities	U = 739 (*p* = 0.6983)
Mean CCM2 of all Mesozoic birds vs mean CCM2 of Sauropodomorphs known from the Tithonian-Maastrichtian	U = 9070 (*p* = 0.01912)
Mean CCM2 of Late Cretaceous birds vs Mean CCM2 of Late Cretaceous sauropodomorphs	U = 678 (*p* = 1×10^−7^)

#### Avian versus sauropodomorph CCM2 values

**Figure 10:**
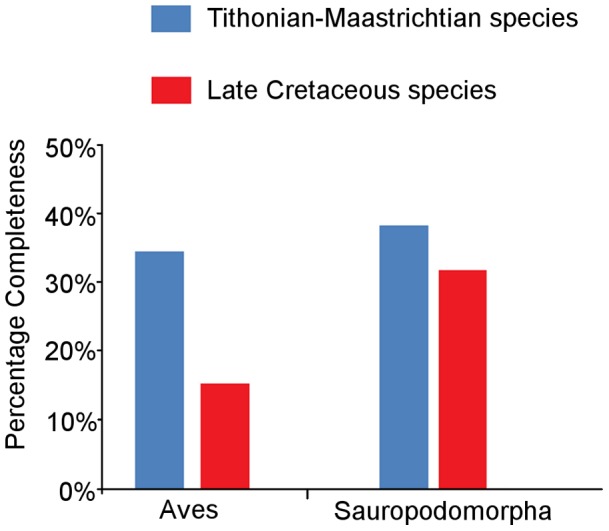
A comparison of the CCM2 scores of Mesozoic bird specimens and that of sauropodomorph specimens [38].

**Figure 11:**
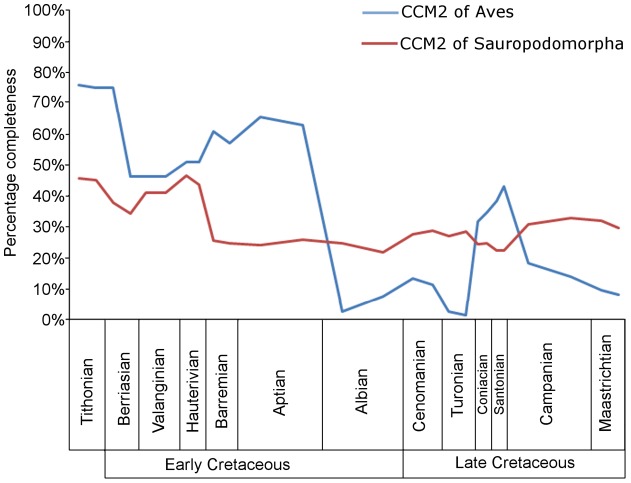
A comparison of the completeness of avian and sauropodomorph specimens. The mean CCM2 scores of all birds (blue curve) and sauropodomorphs (red curve) in each substage from the Tithonian until the late Maastrichtian (data for Sauropodomorpha from reference [38]).

On average, the CCM2 scores for Mesozoic avian species are significantly lower than those of sauropodomorphs [38] when the entire dataset is considered, according to the Mann-Whitney test (Figure 10, Table 3). When we examine Late Cretaceous species alone, this difference between small-bodied birds with delicate skeletons and large-bodied robust sauropods becomes even more marked. When time series of mean CCM2 scores for Mesozoic birds and sauropodomorphs are compared (Figure 11), it is clear that the former series displays a much wider range of values (0–80%) whereas the latter has values that are much more restricted (20–50%). There is no significant correlation between these two time series of mean CCM2 scores (Table 2).

#### Latitude and geographic region

**Figure 12:**
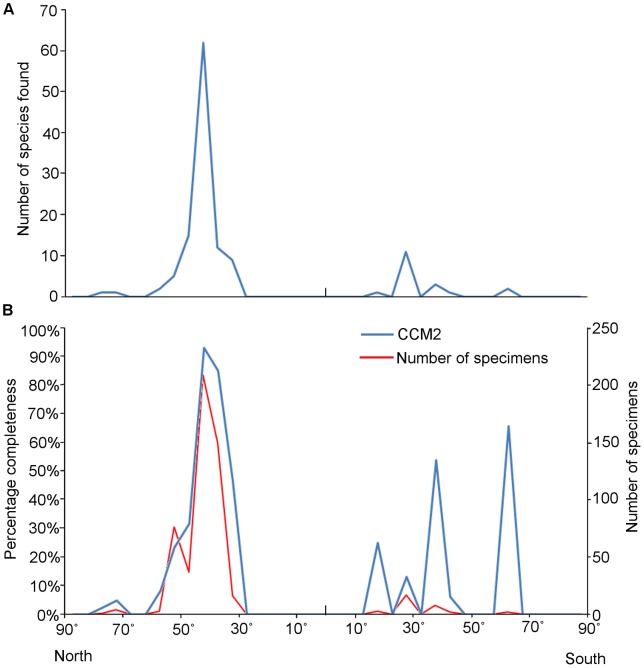
The number and mean CCM2 score of Mesozoic bird species found within modern latitudinal bins. (A) The number of species found in each 5° latitudinal bin; (B) – the mean CCM2 score of all birds (blue curve) found in each latitudinal bin, with the number of specimens (red curve) to indicate sample size.

Mesozoic avian species are most diverse and abundant in the Northern Hemisphere between 30 and 60°N (present day co-ordinates), with the most complete specimens occurring between 40 and 45°N (Figure 12). There is one species (*Canadaga*) known from one locality in the 70–75°N latitudinal bin and another locality in the 75–80°N bin [69], [70], which has a much lower CCM2 score than taxa from the rest of the Northern Hemisphere. To date, no Mesozoic bird species have been found between 30°N and 15°S. The Southern Hemisphere record contains considerable gaps, with no birds found between 20 and 25°, 30 and 35°, 40 and 60°S, and none known from further south than 65°S. The latitudes from which the most Southern Hemisphere species are known are between 25 and 30°S, but the most complete specimens are found between 60 and 65°S. Similar latitudinal biases in the present day distribution of Mesozoic dinosaur fossils, including birds, were noted by Mannion et al. [71].

**Figure 13:**
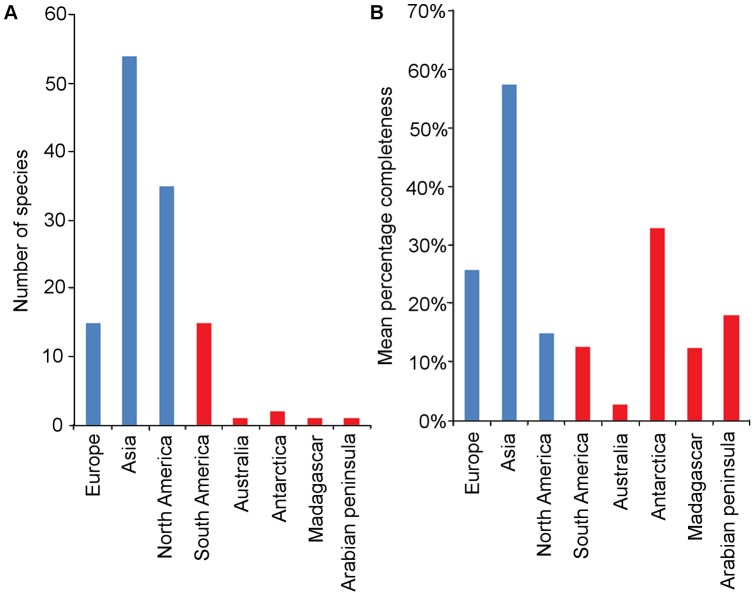
The geographical distribution and completeness (CCM2) of Mesozoic bird species. (A) The number of avian species found in each landmass; (B) the mean CCM2 score of the avian species found on each landmass. Landmasses formerly part of Laurasia are represented by red bars, while those formerly part of Gondwana are represented by blue bars.

Mesozoic avian species are most diverse in Asia (Figure 13). The mean CCM2 value for specimens found in each landmass is also highest in Asia. However, the correspondence between higher species diversity and specimen completeness does not hold for other continents. For example, more species have been found in Europe and North America than in any of the Gondwanan landmasses, yet the mean CCM2 of species known from Antarctica is higher than that of Europe, and the mean CCM2 of species from Antarctica and completeness of the one species from the Arabian Peninsula are higher than that for North America (Figure 13). In Gondwana, most species are known from South America (Australasia, Madagascar and the Arabian Peninsula have each produced only one species, and Antarctica two, whereas South America has produced 15). However, the mean completeness of avian species from Antarctica and the completeness of the one species from the Arabian Peninsula are higher than that of South America (Figure 13). No Mesozoic bird species have been named from Africa or India; although one specimen has been found in Tanzania, it has not been described or named [72].

## Discussion

### Mesozoic bird diversity

The current study presents the most complete and up-to-date analysis of Mesozoic avian diversity, including the first attempt to address potential sampling biases in the avian fossil record via the application of the residual diversity approach. Thus, although the main aim of this paper is to examine aspects of fossil record quality, a reassessment of how avian diversity fluctuated during the Mesozoic is warranted here.

The taxic diversity of Aves in the Mesozoic (Figure 6A) shows a significant positive correlation with the number of theropod-bearing collections from each substage (see Table 2). This suggests that the taxic diversity estimate is probably strongly influenced by collecting effort. Thus, the taxic diversity estimate is probably not an accurate representation of actual Mesozoic diversity. It should be noted that avian taxic diversity does not correlate as closely with the number of dinosaur-bearing formations known from each substage of the Mesozoic, a sampling metric shown to correlate with other dinosaur clades [49], [51], [56], [64]. It seems that in the case of Mesozoic birds, the effects of anthropogenic sampling biases are greater than those of the amount of sedimentary rock known from each time period.

The ‘sampling corrected’ residual diversity curve (Figure 6b) indicates a fall in diversity between the early and late Tithonian, a decline also apparent in the raw taxic diversity curve. In the early Tithonian time bin, three species are known: *Archaeopteryx lithographica* [74] and *Wellnhoferia grandis* [75], [76], both from the Solnhofen Limestone of Bavaria, Germany, and *Shenqiornis mengi*, from the Qiatou Formation of China. In the late Tithonian and early Berriasian, only *Shenquiornis* is present. It should be noted that the Qiatou formation has yet to be reliably dated beyond the fact that it underlies the Yixian Formation [77]. As such, in this dataset (where taxa of uncertain age were included as present in the entire range of substages to which it may have belonged) *Shenqiornis* is present from the Tithonian until the early Barremian.

Although both the raw taxic diversity curve and the residual diversity curve support a drop in avian diversity between the early and late Tithonian this is most likely a result of the early Tithonian Solnhofen Lagerstätte. This area of exceptional preservation will obviously lead to an increase in the diversity inferred from the raw species count, but can also affect the ‘sampling corrected’ diversity, since a relatively small amount of collecting effort can produce high numbers of specimens and new species. Thus, the decline in observed diversity seen during the Tithonian may merely reflect the presence of a Largerstätten in the early Tithonian and the absence of one in the late Tithonian.

During the Early Cretaceous, the residual curve shows a steady increase in avian diversity, which rises to a significant peak in the early Aptian. This radiation includes the appearance and diversification of many of the Mesozoic clades (see Figure 2). The earliest member of the Confuciusornithidae, *Eoconfuciusornis zhengi* [78], appears in the Hauterivian-aged Dabeigou Formation, although a ghost lineage for this family can be inferred at least as far back as the earliest member of the Enantiornithes, in this case *Shenqiornis* (as discussed above, we use the full possible stratigraphic range for this species, extending its range into the Tithonian as a result of the uncertain age of the Qiatou Formation). Otherwise, the enantiornithine *Noguerornis gonzalezi* [79], found in the late Berriasian-Valanginian Montsec Limestone of Spain [80], [81], would be considered the earliest member of this clade, although one study has provided a Hauterivian-early Barremian age for this formation [82]. The oldest members of the Ornithuromorpha appear in the Yixian Formation (Barremian–early Aptian [83]) e.g. *Archaeorhynchus spathula* [84], *Hongshanornis longicresta* [85], *Liaoningornis longidigitris* [86] and *Longicrusavis houi* [87].

At the Aptian/Albian boundary there is a significant drop in avian diversity in both the raw taxic curve and the RDE. Although this apparent extinction affected species numbers, most of the higher avian taxa survived. For example, the Confuciusornithidae had disappeared from the fossil record before this Aptian/Albian event, while the Enantiornithes and the Ornithuromorpha both survived. An alternative explanation for this pattern is that this decrease in diversity is an artefact of uneven sampling of the fossil record. The Aptian record is dominated by the Lagerstätten of the Chinese Yixian and Jiufotang Formations; as discussed above in reference to the Solnhofen Limestone this might have resulted in an increase in both raw taxic diversity and RDE. Thus, this decrease in diversity might be an artefact of, or have been exaggerated by, an overestimation of diversity in the Aptian rather than a true extinction in the Albian. A second possibility is that the perceived extinction is a result of very patchy geographical sampling; of the 23 species known from across this boundary, only two occur outside China. Such a localised record cannot be seen as representative of a worldwide evolutionary event (e.g. [58]).

Avian diversity recovered throughout the Albian and Cenomanian, before a plateau was reached. During the Coniacian and Santonian there is a second significant peak in diversity. This increase is associated with the radiation of the Cretaceous marine birds: the Hesperornithiformes and Ichthyornithiformes. Although Hesperornithiformes and Ichthyornithiformes first appear in the fossil record in the late Albian and the early Turonian respectively [88], [89], they did not become speciose until the Coniacian. This diversity peak was ephemeral, as at the Santonian/Campanian boundary diversity fell to levels lower than that of the late Turonian. As at the Aptian/Albian boundary, this decline does not seem to be accompanied by the extinction of any higher-level taxa; the Enantiornithes and Hesperornithiformes survived until the K/Pg boundary, while the Ichthyornithiformes survived into the Campanian (see figure 2).

During the early Maastrichtian, both the taxic and residual diversity estimates support an increase in diversity. However, many of the species in the Maastrichtian are too fragmentary to assign them to a particular clade, and so it is difficult to assess whether these end-Cretaceous radiations are related to the origination and diversification of new higher taxa. It should be noted that the earliest known unambiguous neornithine bird, the anatoid *Vegavis*, appears in Maastrichtian sediments [30]. This increase in diversity may reflect the diversification of this clade and other neornithine clades for which Maastrichtian ghost lineages may be inferred (see Figure 2). Longrich et al. [22] argued for an extensive radiation of more basal Ornithurae preceding the Cretaceous/Palaeogene extinction. However they noted that the only reliably dated record of Mesozoic birds immediately preceding the extinction is found in North America, and so our interpretations of the fossil record at this time should be treated with caution [22]. The residual diversity curve actually indicates a decline in diversity between the early and late Maastrichtian.

### Comparisons with Fountaine et al.'s study

Fountaine et al. [37] found that no Mesozoic geological stage, with the exception of the Maastrichtian, preserves an overabundance of fragmentary avian material (Figure 5A). In contrast, mean CCM2 values presented here suggest that there is considerably more variation in fossil record quality between the stages, with extremely fragmentary material occurring in the late Berriasian, early Valanginian and several stages of the Late Cretaceous (Figure 3). This difference does not seem to reflect changes caused by the discovery of new species during the past eight years: when the CCM approach is applied to only species used by Fountaine et al., there is very little difference between the two CCM2 time series and they are strongly and positively correlated (Table 2, Figure 4). It seems that, although the 28 new species described since 2003 have improved the average completeness of taxa between the late Tithonian and the Barremian, the overall trend of completeness through time has not been affected. One must also note that the difference in the completeness scores between the complete and pruned datasets in the late Tithonian and early Berriasian is entirely due to the discovery of *Shenquiornis*. In the pruned dataset, no specimens are present in these substages, while in the complete dataset, one very complete specimen is present. Not only should the uncertain date of this specimen be emphasised (see above), but also the effect of the small sample size on our results. Other stages have shown no improvements in the completeness of specimens with the influx of new discoveries, indicating that one or more geological and/or geographical factors might be limiting the quality of specimens from such time periods. Similarly, this result indicates that recently discovered, high quality Mesozoic bird fossils have come from time bins that have previously yielded highly complete taxa. This effect is presumably related to the continuing exploitation of Lagerstätten, such as the Liaoning avian fauna of China, which has produced 15 of the 28 new species discovered since 2003.

If the differences between Fountaine et al.'s study and that presented here do not stem from new discoveries, then they must instead reflect differences in methodology, as is demonstrated by the weaker correlation between the mean CCM2 and the mean completeness grades assigned by Fountaine et al (Figure 5b). The first distinction to note is the difference in temporal resolution: Fountaine et al. [37] calculated the completeness of the species in each stage of the Mesozoic, whereas the current study utilizes substages. Fountaine et al. also did not produce a time series with their data, but instead compared the ratios of the completeness grades in each time period. Assessment of the completeness of species based on either a 1–4 grading scheme or CCM2 results in important differences in interpretation. For example, consider how avian species completeness for the Cenomanian and Santonian are estimated using the two approaches. Fountaine et al. gave all Cenomanian and Santonian bird species a completeness grade of 2 (i.e. each species is represented by an association of a few disarticulated elements). This implies similar fossil record quality in both stages. In contrast, the mean CCM2 scores for the Cenomanian substages are 13.62% and 13.59%, whereas the Santonian substages have mean CCM2 values of 38.89 and 43.72%. Thus, CCM2 indicates a major difference in quality between the two stages. This disparity between estimates of fossil record quality stems from the relatively coarse-grained nature of Fountaine et al.'s grading system, compared to the fine-grained nature of the CCM2. For example, the Cenomanian bird species *Pasquiaornis hardiei* and *P. tankei* are represented by several bones [90], meaning they score a grade of 2 using Fountaine et al.'s method. The CCM2 produces low completeness estimates for these species (8.70% and 14.75% respectively) because almost all available bones are from the same skeletal region (the hindlimb) and several specimens duplicate the same element. Santonian bird species are also represented by collections of disarticulated bones; however, several of these species, such as *Hesperornis regailis*, *H. crassipes*, *Parahesperornis alexi*, *Baptornis advenus* and *Ichthyornis dispar*, are represented by very large collections of bones, covering a much wider range of body regions [89], [91]–[93], and thus many more phylogenetic characters can be scored for each species. This is reflected in a much higher mean CCM2 score for the Santonian. As the method of Fountaine et al. does not take into account differences in the anatomical positions of the known elements, this variation in taxon completeness is not observable in their dataset. Thus, we suggest that the CCM approach is preferable to completeness grading schemes such as those proposed by Fountaine et al. [37] and Benton [47] because the relatively coarse-grained nature of the latter results in failure to detect significant differences in fossil record quality.

Despite the differences noted above, there are some important points of agreement between our results and those of Fountaine et al. [37]. First, both studies support high completeness in the Tithonian (the earliest stage from which birds are known); the mean CCM2 value for this stage is higher than any other in the Mesozoic, and Fountaine et al. were able to give all species a grade of 3 or 4 (i.e. taxa are represented by one or more nearly complete skeletons). This is surprising: it might be expected that the quality of the fossil record would decrease with greater stratigraphic age [94] because older fossils have more time to be eroded, damaged or subducted. The high completeness score of this time period reflects the effect of Lagerstätten: two of the three early Tithonian birds are from Solnhofen in Germany, an area of exceptional preservation [95].

Second, both studies show low completeness scores for the Maastrichtian, the last stage of the Cretaceous. Fountaine et al. gave all species in this time period a grade of 1 or 2, reflecting the lack of nearly complete specimens. Comparably, the mean CCM2 values for the Maastrichtian range from only 7.60% to 9.82%. Again this contradicts the expectation that the youngest time bin should have a better fossil record. However both these studies support the notion that the fossil record's completeness is in fact random with respect to geological age. In order to explain these low mean CCM2 values, we instead need to examine biotic and abiotic factors that might influence completeness.

### Completeness Metrics and Diversity

During the past few decades, there has been a great deal of discussion concerning how uneven sampling of the fossil record might affect the accuracy of palaeobiological studies of taxic diversity [54]. Potential sampling biases include both geological factors (such as temporal fluctuations in the availability of fossiliferous rocks) and anthropogenic factors (such as variation in collecting effort with respect to stratigraphy and/or geographic region). Attempts have been made to measure and ‘correct’ these sampling biases using techniques based on ghost range estimation (e.g. [96]), subsampling (e.g. [97]–[99]), and sampling metrics such as quantification of rock volume or outcrop area, and counts of the numbers of geological formations, localities or collections, per time period (e.g. [54], [73], [100]). Mannion & Upchurch [38] suggested that their completeness metrics might provide an additional sampling metric that captures an aspect of sampling that is ignored by other approaches. Theoretically, time bins with low mean completeness values will produce low numbers of diagnosable species because most specimens are of poor quality and can only be confidently assigned to higher taxa. In contrast, time periods with good preservation will yield many specimens that are rich in diagnostic features, allowing taxonomists to identify numerous new species. If such a mechanism operated in the fossil record, then we would expect completeness metrics such as mean CCM2 to be positively correlated with observed taxic diversity but display little correlation with sampling-corrected diversity estimates. Benton et al. [55] found evidence for this phenomenon; using a previously published [101] assessment of the completeness of Permo-Triassic tetrapods from the South Urals with a four-level grading system, the ‘quality measure’ (number of ‘good’ specimens/total number of specimens) was found to correlate with genus-level diversity. There are, however, factors that might complicate the relationship between completeness metric values and observed taxic diversity in the fossil record. For example, Mannion & Upchurch [38] presented a hypothetical situation in which a time bin with low genuine diversity might have its raw taxic diversity count artificially inflated as a result of poor preservation of specimens. Essentially, the occurrence of fragmentary and largely non-overlapping specimens increases the likelihood that a taxonomist will recognise several diagnosable species based on isolated elements that actually belong to a single species. The extent to which poor preservation results in artificially inflated or artificially lowered estimates of taxic diversity will depend on the attitude of the taxonomists studying the fossil material. Those workers inclined towards taxonomic ‘lumping’ are more likely to underestimate true diversity, whereas those inclined towards taxonomic ‘splitting’ are more likely to overestimate it. This issue will be less problematic when taxonomists work with material from time periods that have yielded generally more complete specimens: such specimens are more likely to display anatomical overlap, making it easier to accurately refer specimens to existing taxa or distinguish them as new taxa.

It is also conceivable that genuine evolutionary events, such as changes in the abundance and/or diversity of a group could influence the completeness of fossils in each time bin. Time periods when species are particularly diverse, abundant and geographically widespread could have an increased probability of preserving highly complete specimens. If such factors operated in the Mesozoic avian fossil record, then we might expect a positive correlation between mean CCM2 scores and the ‘sampling-corrected’ residual diversity estimate.

In this context, it is interesting to note that the current study found a significant positive correlation between mean CCM2 and both raw taxic diversity and the residual diversity curve, but with the latter correlation the stronger of the two. The strong positive correlation with residual diversity, which should more closely represent the genuine diversity of Aves in the Mesozoic, suggests that their abundance and diversity has affected the probability of more complete specimens entering the fossil record and surviving to the present day. The correlation with the raw taxic diversity curve does suggest that there is also an influence of specimen completeness on the ability of taxonomists to recognise new taxa, as suggested by Mannion and Upchurch [38]. However, the effect of completeness on observed diversity seems to be less marked than the impact of genuine changes in diversity and/or abundance on completeness.

### Controls on the Completeness of the Avian Fossil Record

#### Sea level

The impact of sea level change on fossil record quality and taxic diversity is complex and controversial. Fluctuations in sea level clearly have the potential to change preservation rates in particular environments. Some studies have found positive correlations between sea level and the raw taxic diversity of marine organisms, suggesting that increased formation and preservation of coastal deposits have resulted in a higher quality fossil record. A similar argument has been made for some terrestrial groups on the basis that higher sea level increases the preservation of terrestrial taxa whose remains are washed into deltas, estuaries, lagoons etc. [102]. Conversely, a negative correlation between sea level and the quality of the terrestrial fossil record is also supported (e.g. [38]), because higher sea level reduces the available land area and so decreases the amount of terrestrial sedimentary rock [54], [103]. The relationship between sea level and fossil record quality is further complicated by ‘common cause’ hypotheses which argue that rises in sea level simultaneously promote increased preservation of fossils and increases in diversity (the latter mediated by factors such as the radiation of groups living in the expanded near shore environments) [60], [104], [105]. On land, common cause could take the form of either a positive or a negative correlation between sea level and taxic diversity. One possibility is that rises in sea level result in fragmentation of terrestrial land areas and habitats, promoting an increase in diversity [57], [106], [107]. Alternatively, higher sea level reduces available land area and, according to the species-area relationship, this should result in decreases in the diversity of terrestrial taxa [104], [108], [109]. However, Butler et al. [53] demonstrated that sea level does not correlate with either the raw taxic diversity of dinosaurs or sampling metrics once time series data are detrended and the effects of autocorrelation are taken into account (more detailed discussion of the role of sea level on observed diversity in the fossil record, evolutionary radiations and extinctions, and sampling biases, can be found in references [53], [58], [62], [104]).

Mannion & Upchurch [38] noted that sea level varies inversely with the completeness of sauropodomorph specimens during the Cretaceous. This suggests that a high sea level, while not affecting dinosaur diversity, might affect the preservation potential of terrestrial organisms. Here, our results show no correlation between the mean CCM2 scores of birds and the sea level curve [53]. This could be because Cretaceous birds were not restricted to terrestrial environments, unlike sauropodomorphs. Mannion & Upchurch [110] demonstrated that only 0.01% of the sauropodomorph fossil record comes from marine deposits, and such fossils almost certainly represent rare instances where sauropod carcasses were washed out to sea. In contrast, several groups of Cretaceous bird flourished in marine environments, including the Hesperornithiformes (flightless aquatic birds) and Ichthyornithiformes (thought to be the ecological equivalents of modern gulls and terns) [89]. As such, fluctuations in sea level would not necessarily change the total area available for the preservation of birds: instead, they might merely shift preservation rates in favour of birds from particular habitats. Thus, at times of high sea level marine birds might have a higher preservation potential, whereas at times of low sea level terrestrial birds might be better preserved. If correct, the wider ecological range of Cretaceous birds might explain why their mean CCM2 values do not correlate with sea level.

#### Habitat and depositional environment

Another factor that might affect the quality of avian fossils is the environment of the locality in which they are preserved. Preservation should be best in low energy environments, where carcasses are less likely to be damaged by post-mortem transportation and/or erosion. Such environments include lakes, river floodplains, deltas and lagoons. The results of the analysis of the completeness of birds found in localities representing different environments (Figure 8) indicate that birds from fluviolacustrine localities are more completely preserved than those from marine and other terrestrial localities (mean CCM2 46.51%). The birds from marine and non-fluviolacustrine terrestrial localities had similar mean CCM2 values (24.97% and 30.87% respectively), significantly lower according to the Mann-Whitney U test (Table 3), presumably reflecting the fact that fossils in both are at greater risk of erosion. Carcasses in fluvial environments are also at risk of erosion, but lacustrine, floodplain and deltaic environments, with sluggish water, are expected to yield more complete specimens.

Given the differences between the mean CCM2 values of the three depositional categories, it is possible that the number of localities in a particular environment controls the completeness of avian fossils in each time period. For example, it is interesting to note that during the Albian, Cenomanian, Turonian and the late Maastrichtian (times where the mean CCM2 is particularly low) none of the bird specimens come from fluviolacustrine environments. To investigate this possibility, the mean CCM2 values were compared to the number of fluviolacustrine bird-bearing localities per time bin (Figure 9). The Spearman's rank and Kendall's tau values indicate that there is no significant correlation between these two time series (Table 2). Therefore, although depositional environment does seem to affect the quality of the avian fossil record, it is not the dominant control.

#### Geographical Controls

Sampling of the fossil record varies not only across stratigraphic intervals, but also geographically. These spatial sampling biases result from both geological factors (such as how much fossiliferous rock of a given age occurs on each continent or within each latitudinal zone) and anthropogenic factors (such as the number of active palaeobiological researchers supported by different countries). For example, dinosaur diversity is dominated by species from North America and Asia [111], not necessarily because dinosaurs were more speciose in these regions, but because of greater collecting effort combined with exposure of larger tracts of fossiliferous rock. Mesozoic avian diversity follows a similar pattern, with the northern landmasses which made up the Laurasian supercontinent (Asia, North America and Europe) yielding considerably more species than the southern continents that formed Gondwana (South America, Australia, Madagascar, the Arabian Peninsula and Antarctica) (Figure 11). For example, Africa and the Indian subcontinent have produced no avian species at all, whereas Asia is responsible for 44.35% of the 124 valid Mesozoic bird species in our dataset. Laurasian landmasses as a whole account for 85.48% of Mesozoic bird species and 71.63% of species from the Late Cretaceous.

In contrast, geographical variation in mean CCM2 values does not display the same regional skews. Although Asian birds are both more diverse and more complete than those from other continents (Figure 13), this probably reflects the exceptional preservation of the Chinese specimens (i.e., a Lagerstätten effect produced by exceptional deposits such as the Yixian Formation). Aside from this skew caused by Chinese fossils, there is no correlation between the number of avian species known from a continent and the mean CCM2 score for those species. For example, the birds of the Arabian Peninsula and Antarctica (very under-sampled landmasses where a few avian fossils have been found only recently) have a higher mean CCM2 score than those found in North America, where Mesozoic bird fossils are abundant and have been known since the 1800s (Figure 13). The high completeness of the birds found in these as-yet unproductive areas could be an indication that the low number of species and specimens found is not caused by problems with preservation in these areas, but instead reflects the lack of sampling. The low number of specimens from these areas should be noted; it is possible that the high completeness of the bird species found here results from the random possibility of the first few specimens being found there being of high completeness. However, this does still indicate that the preservation of high-quality specimens is possible in these areas.

The number of taxa found within modern day 5°latitudinal bins is plotted in Figure 11A, while mean CCM2 values are plotted against latitude in Figure 11B. Sampling of the Southern Hemisphere is clearly poorer than that of the Northern Hemisphere. There are more empty latitudinal bins in the Southern Hemisphere (13 bins, compared to 10 in the Northern Hemisphere). No latitudinal bin in the Southern Hemisphere contains more than seven species. In contrast, in the Northern Hemisphere, four latitudinal bins have produced more than this number of taxa. Between 40 and 45°N, a total of 62 species have been recovered. However, the mean CCM2 values for those Southern Hemisphere latitudinal bins that have yielded Mesozoic birds are not substantially lower than the mean CCM2 values for equivalent bins in the Northern Hemisphere (typically between 30–60° north of the Equator) (Figure 11). These latitudinal analyses indicate regions which have potential to yield high quality specimens in the future. Although only two taxa have been recovered from high latitudes in the Southern Hemisphere (those further south than 50°S), the specimens are well preserved: *Vegavis iaai* and *Polarornis gregorii* have CCM2 scores of 40.00% and 25.65% respectively. Despite the logistical difficulties associated with fieldwork in Antarctica, our results suggest that this continent could provide a great deal of further information on avian evolution. In contrast, the tropical regions between 25°N and 20°S have produced no Mesozoic bird taxa. Finally, although the Northern Hemisphere has been well sampled in mid-latitudes, there is very little avian material of high quality from within the Arctic Circle, again presumably resulting from the difficulties of working in that environment. However, *Canadaga arctica*, known from two localities in northern Canada (between 70 and 80°N) [69], [70], indicates that there is future potential to sample specimens from a far-north avian fauna.

#### Taphonomic effects: body size and skeletal robustness

**Figure 14:**
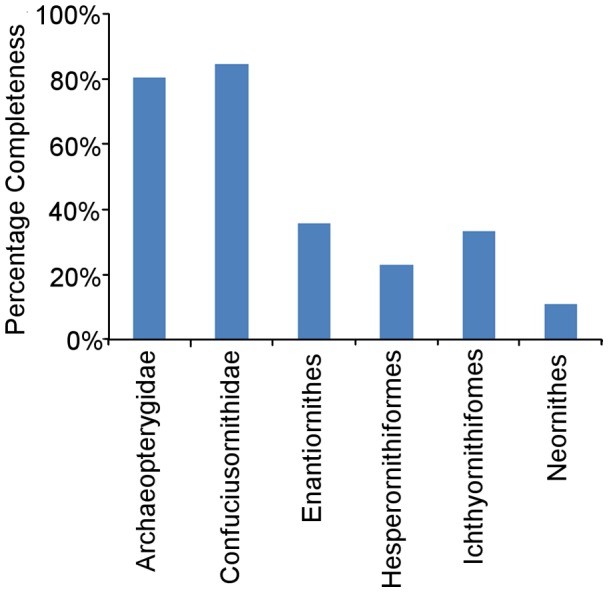
The mean CCM2 scores for birds assigned to the major Mesozoic avian clades.

Analysis of the mean CCM 2 data for Sauropodomorpha in each substage of the Mesozoic (figure 14) shows that there is no statistically significant correlation between the completeness of their record and that of contemporaneous Aves. This suggests that the completeness of large, robust animals is controlled by different factors to small animals with fragile skeletons (see also studies of the disarticulation of extant animals e.g. [112]). This is not surprising considering the different ways in which members of these two clades are preserved and discovered. Many of the most complete avian fossils are discovered as part and counterpart, whereby a block is split, revealing a flattened skeleton on one plate and an imprint on the other. Although this produces exceptional preservation of small animals, including soft tissues such as feathers, it cannot preserve complete skeletons of animals as large as sauropodomorphs. Therefore, a time period containing geological formations suitable for this preservation mode would lead to a large increase in the completeness of bird fossils, but have little effect on the completeness of sauropodomorphs. Conversely, time periods which include a preponderance of geological formations representing high energy environments, would generally yield better quality sauropodomorph material than avian material. Finally, as noted above, birds are often preserved in marine environments, whereas very few sauropodomorph fossils occur in such deposits. Time periods preserving numerous formations composed of marine deposits might therefore result in an increase in the completeness of bird specimens, but would make very little difference to the sauropodomorph record. Thus, there is little reason to expect that the avian and sauropodomorph mean CCM2 scores should correlate.

The mean CCM2 scores of sauropodomorphs during the Cretaceous show less variation than those of birds. Apart from a peak in the Hauterivian of 47.33%, the completeness of sauropod species varies between a narrow range of 22.71% and 33.50% [38]. In contrast, the mean CCM2 scores for birds range from 1.53% to 75.72% over the same time period. This observation probably reflects differences in the factors that control the completeness of avian and sauropod specimens. In particular, the differences in body size and robustness of birds and sauropodomorphs might explain the greater variation in mean CCM2 values for the former and the lower variation for the latter. Depending on the precise geological setting, it is perhaps easier to utterly destroy an avian carcass during transportation or via erosion than a sauropodomorph carcass: however it is also easier to rapidly and completely bury a small skeleton (resulting in high completeness scores), whereas it is much less likely that a 20–30 m long sauropod skeleton will be preserved intact. This suggests that Largerstätten effects on observed diversity are likely to be far more severe for small delicate organisms such as birds than for large robust ones such as sauropods.

As noted in the ‘Introduction’ above, some molecular clock studies [10]–[13] have explained the absence of well-preserved Cretaceous neornithines in terms of the low preservation potential of birds. Avian species today, and in the past, are typically small-bodied and lightly built because of the constraints imposed by powered flight. We might expect, therefore, that bird carcasses would be particularly susceptible to damage and destruction during post-mortem transportation and erosion and consequently should have a poorer fossil record than larger and/or more robust contemporaneous taxa. The simple completeness metric for tetrapods [35], however, suggests that the record of small-bodied animals is no less complete than that of larger ones. This prediction can be tested by comparing the mean CCM2 values for birds and sauropodomorphs. In fact, when taking into account the entire period of the Mesozoic from which birds are known (from the Tithonian– Maastrichtian), despite the mean CCM2 of birds being only 4% less than that of sauropodomorphs (Figure 10), the difference between the medians is significant, as shown by the Mann-Whitney U-test (Table 3). When Late Cretaceous species alone are evaluated, the mean CCM2 score for birds is 16% less than that of sauropodomorphs (Figure 10, Table 3). Thus, contrary to the simple completeness metric results for tetrapods [35], CCM2 values suggest that larger and more robust organisms are better preserved than small delicate ones, at least in Dinosauria. Moreover, whatever factors are driving the differential preservation of small, delicate animals and large, robust ones, these factors do not appear to be constant through time.

### The Origin of Neornithes

Claims that Neornithes are genuinely absent prior to the Maastrichtian (because they originated just before and radiated after the K/Pg mass extinction [16], [20], [31]) can only be supported if the Cretaceous avian fossil record is well sampled temporally, spatially and in terms of specimen/taxon completeness. Fountaine et al. [37] argued that (with the exception of taxa of Maastrichtian age) Mesozoic birds are represented by high enough quality material to infer a genuine absence of Neornithes during the Cretaceous. However, this uniformity of relatively high quality preservation throughout much of the Cretaceous is not supported by our analyses. Mean CCM2 values display considerably more variation between time bins than do the completeness grades of Fountaine et al. We suggest that the relatively low variation in completeness scores recovered by Fountaine et al is an artefact generated by the application of a very coarse-grained completeness metric: the more sensitive mean CCM2 scores support the view that species completeness varied considerably during the Mesozoic, with peaks reflecting the occurrence of Lagerstätten such as Solnhofen and the Yixian Formation. Such Lagerstätten are absent from the Late Cretaceous, resulting in relatively low mean CCM2 values for most of this period. All stages of the Late Cretaceous have a mean CCM2 score of less than 19%, with the exception of the Santonian and Coniacian (Figure 3). The avian mean CCM2 scores are much lower than those produced for sauropodomorphs [38] for all stages of the Late Cretaceous, again with the exception of the Coniacian and the Santonian (Figure 14). The mean completeness of birds from the Late Cretaceous is considerably lower than that of all Mesozoic birds, and less than that of Late Cretaceous sauropodomorphs (Figure 10), indicating worse preservation of small delicate animals at this time.

The Cretaceous avian fossil record is also very patchy in terms of spatial sampling. This is particularly noticeable with regard to the tropics and high latitudes, with the majority of Mesozoic avian species (84%) being found between 30 and 60°North of the Equator (Figure 11). There are also entire regions, such as Africa and India, which have produced no diagnostic avian material. The relatively poor sampling of Gondwanan continents is particularly noteworthy given that some biogeographic analyses (e.g. [14]) have suggested that Neornithes originated in the Southern Hemisphere. Clearly, it is unrealistic to claim that the Cretaceous fossil record is adequate for determining the genuine absence of Neornithes if it transpires that this clade originated and initially radiated at high latitudes or in the tropics, or in regions such as Africa.

It should be noted that it is in the poorly sampled region of Antarctica that the two most complete pre-Palaeogene putative neornithines have been found. *Polarornis gregorii* from the Lopez de Bertodano Formation of Antarctica (late Campanian-Maastrichtian) [113] is well preserved, including a nearly complete skull, several vertebrae, a sternum and portions of the hindlimb [2] (CCM2 score  = 25.65%). Chatterjee [114] assigned this species to the neornithine family Gaviidae (loons). If both the age of the site and the affinities of the specimen are correct, this places the origin of the Gaviidae at a time which correlates well with the molecular clock study of Cooper & Penny [12]. However, the stratigraphic age of the Lopez de Bertodano Formation has yet to be verified [18], and the assignment of *Polarornis* to the loons was based on overall similarity rather than cladistic analysis. Of the six characters used to support the relationship with Gaviidae, five have been found in more basal birds and even non-avian theropod dinosaurs [18]. Thus, until cladistic analysis is applied, placement of *Polarornis* in Neornithes remains questionable. In contrast, *Vegavis iaai*, from the Maastrichtian of Antarctica, is known from a nearly complete postcranial skeleton (CCM2 score  = 40.00%), and has been subjected to phylogenetic analysis which placed it within the Anatoidea [30]. If this identification is correct, then a derived neornithine lineage was present in the latest Cretaceous, implying an earlier origin for Neornithes as a whole. This discovery indicates the possibility that Antarctica has more information to offer on this particular debate. The recent discovery of a possible neornithine carpometacarpus in Argentina [27] highlights the potential for future discoveries elsewhere in the Southern Hemisphere.

Comparisons of the CCM2 values for putative Cretaceous Neornithes versus other avian clades indicate that the former group are particularly poorly preserved (Figure 14). There are two possible explanations for the differences in completeness of neornithine and non-neornithine birds:

1. Fragmentary preservation leads to ambiguity of identification. It is conceivable that more complete Cretaceous bird specimens provide enough anatomical detail for them to be confidently assigned to non-neornithine lineages. The more fragmentary specimens assigned to Neornithes might actually belong to non-neornithines, but their poor preservation generates more ambiguity in their identification. For example, if some Cretaceous non-neornithines convergently acquired some derived character states that also occur in true Neornithes, then these convergences might only be detected when specimens are well preserved (thus providing additional character data that contradicts interpretation of these features as synapomorphies of the Neornithes). Conversely, convergence might go undetected when only fragmentary specimens are available.
2. The effects of abundance and habitat. A second possibility is that, during the Cretaceous, Neornithes were generally less abundant than other bird groups [37]. Lower numbers of individual birds might decrease the probability that some well-preserved specimens will successfully survive post-mortem transportation and fossilization, as well as increasing the chances of discovery. Similarly, it is also possible that the first Neornithes lived in habitats that are less likely to preserve highly complete specimens. For example, our results demonstrate that Mesozoic birds living in fluviolacustrine environments tend to be more complete than those living in marine or other terrestrial ones. If Neornithes radiated initially in other niches, their fossil record could be substantially poorer than that of non-neornithines occupying fluvial and lacustrine habitats.

In short, fossil evidence for the occurrence of Early or early Late Cretaceous neornithines, as predicted by molecular clocks, remains elusive. The Late Cretaceous avian fossil record is particularly fragmentary because of an absence of suitable Lagerstätten, and there are several large geographical regions, wide latitudinal zones and portions of the stratigraphic record that are poorly sampled or have yielded no avian fossils of any kind. Thus, it is premature for palaeobiologists to claim that the Cretaceous avian fossil record is sufficiently well sampled to determine that Neornithes were genuinely absent until after or shortly before the K/Pg mass extinction.

### Conclusions

Although specimen/taxon completeness represents only one aspect of fossil record quality, it is a significant one because of its potential relationship to constraints on the accurate recognition of valid taxonomic units. To date, analyses of fossil record quality based on estimates of specimen/taxon completeness have been restricted to studies of dinosaurs (including birds) [37], [38], [42], Permo-Triassic tetrapods [55], [101] and echinoids [115]. Pioneering studies in this field utilised simple grading schemes, but these are problematic because of the arbitrary boundaries between grades and the coarse-grained picture of completeness they generate. A completeness metric based on scorable morphological characters, such as our character completeness metric (CCM2), circumvents both of these problems. Such completeness metrics can be used to generate estimates of fossil record quality, which in turn can be compared to various aspects of sampling, geological and environmental factors, diversity and other evolutionary events. There is growing interest and concern regarding the influence of Lagerstätten on diversity patterns observed directly in the fossil record and on supposedly ‘sampling-corrected’ estimates of palaeodiversity [59]: completeness metrics should make an important contribution to this field in the future given their ability to identify time periods where specimen/taxon completeness is unusually high or low.

The Mesozoic fossil record of birds has clearly been strongly influenced by uneven sampling. This is manifested in the positive correlations between sampling metrics and raw taxic diversity, the significant fluctuations in mean CCM2 values through time, and the very patchy spatial and temporal distribution of taxa. Mesozoic bird specimens are best preserved in conditions such as low energy lacustrine environments, where post-mortem transport and erosion are minimal. These conditions characterise deposits such as those of Solnhofen and the Yixian Formation that represent Lagerstätten. The avian fossil record is not noticeably poorer than that of large bodied robust sauropods except in the Late Cretaceous. The completeness of birds in different time bins is significantly more variable than that of sauropods, reflecting the possibility that bird carcasses are easier to destroy entirely but also easier to bury whole, than those of sauropods. It will be interesting to see if future studies of the completeness of other small and large bodied vertebrates conform to the same patterns. If these patterns do hold, it suggests that Lagerstätten effects on reconstructions of palaeodiversity are likely to be more significant for small delicate organisms than for large robust ones.

Avian diversity, indicated by the ‘corrected’ residual diversity estimate, increased steadily between the Berriasian and the Aptian. Between the Aptian and the Albian there appears to have been a large extinction, although this may be an artefact of sampling, in particular the effect of Lagerstätten. Diversity recovered during the Albian and Cenomanian, and then plateaued during the Turonian. The diversification of the Hesperornithiformes and Ichthyornithiformes during the Coniacian and Santonian led to a peak in the number of species in the Mesozoic. Diversity fell during the Campanian, before rising again in the Maastrichtian.

Both the residual diversity and the taxic diversity curves correlate significantly and positively with the CCM2; however, the correlation with the residual diversity curve is stronger. This suggests that biotic factors such as fluctuations in diversity, abundance and geographic range, have affected the frequency with which bird carcasses enter depositional environments where high quality preservation is possible. The significant, albeit weaker, correlation with the raw taxic diversity curve indicates that the completeness of specimens may place constraints on the ability of taxonomists to recognise new species and/or identify specimens as members of already known species.

The debate concerning the origin of Neornithes before or after the end-Cretaceous mass extinction cannot be settled by an analysis of fossil record quality alone. However, such an analysis can provide a valuable perspective on claims and counter-claims regarding the probability that Neornithes were genuinely absent in the pre-Maastrichtian Cretaceous or were present but have not been found yet. Our results indicate that most of the Late Cretaceous (not just the Maastrichtian as proposed by Fountaine et al. [37]) fossil record of birds is characterised by numerous highly fragmentary specimens. If, as has been proposed by several molecular and biogeographic studies, Neornithes originated in the Late Cretaceous in the Southern Hemisphere (especially at high latitudes), then it is quite plausible that we would not see any unambiguous Cretaceous neornithines in the currently available fossil record. A compelling case for the absence of pre-Maastrichtian Neornithes can be made only after significant gaps in the record (e.g. Africa) have been filled via the discovery of well-preserved non-neornithine birds.

The discrepancy between divergence time estimates based on molecular clocks and direct examination of the fossil record is not unique to the debate over neornithine origins. A very similar discussion has occurred in recent years concerning the origin of placental mammals before or after the K/Pg boundary (e.g. [20], [116]). It is hoped that the current study will stimulate further interest in the application of completeness metrics and other measures of fossil record quality in order to evaluate the likelihood that ‘absence of evidence’ might, or might not, also be ‘evidence of absence’.

## Supporting Information

Table S1 **The genus, species, specimen number, geographical information, stratigraphic and environmental information and CCM2 completeness values for all species used in this analysis.** Rows with ‘Combined’ as the specimen number indicate the combined completeness of all specimens of that species in that time slice. Rows in bold type include the values which contribute to the CCM2 curve.
(XLS)

Table S2 **Character list, with characters organised by the region to which they refer.**
(XLS)

Table S3 **The percentage of characters referring to each bone in the skeleton.**
(XLS)
